# A novel larval diet interacts with nutritional stress to modify juvenile behaviors and glucocorticoid responses

**DOI:** 10.1002/ece3.7860

**Published:** 2021-07-28

**Authors:** Cristina C. Ledón‐Rettig, Sarah R. Lagon

**Affiliations:** ^1^ Indiana University at Bloomington Bloomington IN USA

**Keywords:** carryover effects, catch‐up growth, compensatory growth, corticosterone, stress axis, trophic polyphenism

## Abstract

Developmental plasticity can allow the exploitation of alternative diets. While such flexibility during early life is often adaptive, it can leave a legacy in later life that alters the overall health and fitness of an individual. Species of the spadefoot toad genus *Spea* are uniquely poised to address such carryover effects because their larvae can consume drastically different diets: their ancestral diet of detritus or a derived shrimp diet. Here, we use *Spea* *bombifrons* to assess the effects of developmental plasticity in response to larval diet type and nutritional stress on juvenile behaviors and stress axis reactivity. We find that, in an open‐field assay, juveniles fed shrimp as larvae have longer latencies to move, avoid prey items more often, and have poorer prey‐capture abilities. While juveniles fed shrimp as larvae are more exploratory, this effect disappears if they also experienced a temporary nutritional stressor during early life. The larval shrimp diet additionally impairs juvenile jumping performance. Finally, larvae that were fed shrimp under normal nutritional conditions produce juveniles with higher overall glucocorticoid levels, and larvae that were fed shrimp and experienced a temporary nutritional stressor produce juveniles with higher stress‐induced glucocorticoid levels. Thus, while it has been demonstrated that consuming the novel, alternative diet can be adaptive for larvae in nature, doing so has marked effects on juvenile phenotypes that may recalibrate an individual's overall fitness. Given that organisms often utilize diverse diets in nature, our study underscores the importance of considering how diet type interacts with early‐life nutritional adversity to influence subsequent life stages.

## Graphical abstract

In this study, we assess how early‐life environmental conditions influence fitness‐related traits at future life stages. We specifically use spadefoot toads to understand how their ancestral or novel larval diet type interacts with nutritional stress during early life to shape juvenile phenotypes. We find that the novel diet and temporary nutritional stress generate lasting effects on juvenile behaviors and the regulation of stress hormones.

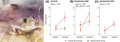

## 1 INTRODUCTION

Early‐life conditions often shape adult behaviors, morphologies, and physiologies. These environmental legacies—often referred to as “carryover effects”—can, in turn, influence the health and fitness of individuals (Metcalfe & Monaghan, 2001; Monaghan, 2008). Thus, while early‐life plastic responses can be shaped by selection to promote adaptive outcomes for developing individuals, the ultimate fitness of these individuals cannot be reckoned until adult life (Moore & Martin, 2019).

Such adult consequences have been measured for plastic responses to several kinds of early‐life adversity (e.g., maternal separation; Feng et al., 2011), as well as the accumulation of different stressors (e.g., Tung et al., 2016). Perhaps the most well‐studied form of early‐life adversity is nutritional stress (Gluckman et al., 2005). Nutritional deficits during development have specifically been shown to modify adult stress axis reactivities (Hu et al., 2008; Pravosudov & Kitaysky, 2006; Warne & Crespi, 2015) and behaviors (Bouchard et al., 2016; Krause et al., 2009, 2011) across taxa. However, it is rarely considered whether the lasting effects of nutritional stress depend on the type of diet being consumed, where diet type may describe variation in resources that require differences in effort, behaviors, morphologies, or physiologies to consume (Futuyma & Moreno, 1988). This is relevant for many ecological systems in which varied or alternative diets can be used. However, assessing whether there is an interaction between nutritional stress and diet type may be challenging in organismal systems where diet composition is continuous in nature. Thus, species that exhibit trophic polyphenism—specifically those that exploit alternate food resources—might be useful in investigating relationships between diet amount and diet type.

Tadpoles of American spadefoot toads (Scaphiopodidae) are uniquely poised to analyze the relationship between early‐life diet type, nutritional stress, and adult outcomes. Larvae of several species in this family are capable of consuming live macroscopic prey in addition to their ancestral diet of detritus (Bragg, 1964; Ledón‐Rettig et al., 2008, 2010; Levis et al., 2020). Notably, those in the genus *Spea* have evolved a trophic polyphenism: In nature, they often occur either as an omnivore morph that specializes on a diet of detritus or, alternatively, as a carnivore morph that specializes on a novel shrimp diet (Pomeroy, 1981). By exploiting this shrimp resource, *Spea* tadpoles reduce competition and increase their chances of attaining the minimum size and stage necessary to metamorphose (Martin & Pfennig, 2009, 2010; Morey & Reznick, 2000; Wilbur & Collins, 1973) before the ephemeral ponds in which they develop evaporate. Although carnivore morphs survive better in fast‐drying ponds, they are also more likely to die immediately postmetamorphosis (Pfennig, 1992; but see de la Serna Buzon et al., 2020), suggesting that utilizing the novel diet comes at a cost later in life. However, it is unclear whether postmetamorphic spadefoots derived from larvae that consumed shrimp are affected in other ways beyond this initial challenge.

Alongside the intense competition they experience, *Spea* tadpoles commonly cope with nutritional stress. The *alleviation* of nutritional stress is also a common experience for tadpoles since competition for food is reduced as other tadpoles metamorphose or are eaten by predators (Burraco et al., 2017). Nutritional restriction that is followed by a sudden abundance of resources can result in compensatory or catch‐up growth (Jobling, 2010); in the former, growth is accelerated after conditions return to normal and in the latter, the developmental growth period is extended to achieve a normal growth outcome. Both types of altered growth patterns have been demonstrated to have detrimental effects on fitness, even relative to permanent nutritional restriction (reviewed in Metcalfe & Monaghan, 2001), for instance, through reduced survival (Birkhead et al., 1999; Lee et al., 2016; Ozanne & Hales, 2004), impaired cognition (Fisher et al., 2006), or delayed reproductive maturity (Morgan & Metcalfe, 2001). Although both compensatory and catch‐up growth have been well‐documented in amphibians (Dahl et al., 2012; Hector et al., 2012; Hu et al., 2008; Orizaola et al., 2014; Warne & Crespi, 2015), the interactions between catch‐up or compensatory growth and diet type have not been addressed.

Using *Spea bombifrons* (the plains spadefoot toad), we sought a more nuanced understanding of how dietary variation and nutritional stress during larval life influence—either independently or interactively—postmetamorphic phenotypes. We specifically compared behaviors related to shyness and boldness (sensu Koolhaas et al., 1999), jumping abilities, and hypothalamic–pituitary–interrenal (HPI) axis reactivity of juveniles that experienced different diet types and amounts of nutritional stress as larvae. Our measures of behavior and jumping performance were chosen as these might influence an individual's success in securing food or evading predators, while the stress (i.e., HPI) axis was evaluated because it is involved with an individual's energy balance and general physiological response to environmental stressors (Dantzer et al., 2014), all of which are likely important for fitness.

## 2 METHODS

### 2.1 Breeding and microcosm setup

Adult frogs were collected from the field during the summer of 2018 from Willcox, Arizona, and transported to Indiana University. All animals were collected with the permission of a scientific collecting permit from the state of Arizona, and all animals and experiments in this study were approved by the Bloomington Indiana University Institutional Animal Care and Use Committee (IACUC protocol #18‐011‐7). The adult frogs were housed in a temperature‐controlled room set to a 12‐our reverse light:dark cycle and kept at approximately 25.7℃ (details of the room's monthly temperatures are provided in Table S1). All larval and juvenile maintenance and experimentation for the study described below occurred in this room. In April of 2020, mating pairs were selected based on similarity of weight. Breeding tanks were filled with aged water. Adult breeding pairs were injected with luteinizing hormone releasing hormone (LHRH; GenScript RP11937) and allowed to mate overnight; females and males were given approximately 12 and 6 µl of (1 µg/100 µl) hormone, respectively. Eggs were left to incubate and hatch over the following 48 hr. Newly hatched tadpoles from one clutch were randomly selected and singly transferred to 198 individual plastic microcosms (18 × 11 × 14 cm) filled with 800 ml aged water.

### 2.2 Experimental design

**FIGURE 1:**
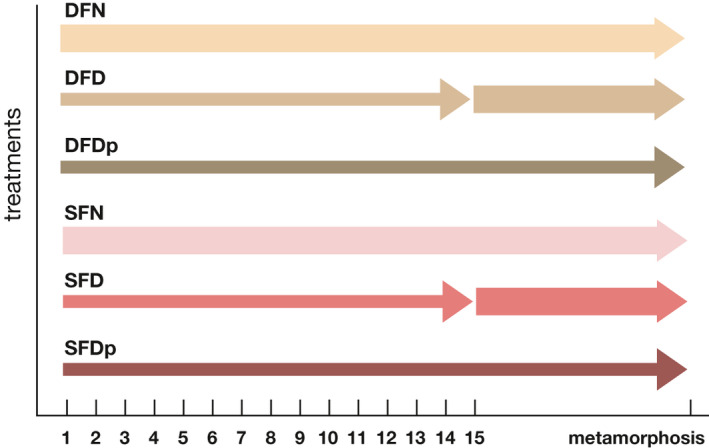
Experimental design. For the first 15 days of feeding, animals were fed either a full (“normal”; thick arrows) or halved (“deficient”, thin arrows) diet. Subsequently, those in the temporarily deficient treatments were transitioned to a normal diet, whereas those in the permanently deficient groups were maintained on a deficient diet. Animals were maintained on these diets until metamorphosis, at which point all individuals were fed a standard cricket diet (DFN, normal detritus‐fed; DFD, temporarily deficient detritus‐fed; DFDp, permanently deficient detritus‐fed; SFN, normal shrimp‐fed; SFD, temporarily deficient shrimp‐fed; SFDp, permanently deficient shrimp‐fed)

To characterize the effects of differing diets and nutritional stress, we used a full factorial design crossing two diet types, detritus and shrimp, with three nutritional amounts: normal nutritional conditions, a temporary nutritional deficit followed by normal conditions, and permanently deficient nutritional conditions (Figure 1). This design resulted in the following six treatments, each with 33 replicates: shrimp‐fed normal (SFN); shrimp‐fed temporarily deficient (SFD); shrimp‐fed permanent deficient (SFDp); detritus‐fed normal (DFN); detritus‐fed temporarily deficient (DFD); and detritus‐fed permanent deficient (DFDp). Individuals in the detritus groups were fed Hikari (Hikari USA; protein 25% fat 4%, fiber 5%) Cichlid Staple pellet fish food (hereafter, detritus), which resembles the detritus found in *S. bombifrons*' natural ponds (Pfennig et al., 1991). In their natal ponds, *S. bombifrons* that specialize on detritus consume decaying plant and animal material as well as algae, and the cichlid pellets include proxies for these different components (e.g., wheat, fish, and seaweed). Individuals in the shrimp treatment were fed *Artemia*, which resemble the shrimp that *S. bombifrons* feeds on in nature (Pomeroy, 1981). Treatments were randomized and interspersed across two racks in the same room.

The administration of diet treatments began immediately after tadpoles were transferred to their microcosms; at the time individuals were transferred, they no longer had external gills and would be prepared to eat within the following 12 hr. The detritus and shrimp treatments were delivered to each microcosm with microscoops and plastic transfer pipettes, respectively. Individuals in the normal nutritional treatments were given an amount of food sufficient for ad libitum feeding, whereas individuals in the temporarily deficient and permanently deficient treatments received half as much food as the normal treatments. Food provisions were incrementally increased as tadpoles grew and their nutritional requirements increased. After 15 days, the temporarily deficient groups were provided the same measures of food for ad libitum feeding for the duration of the larval period, while the permanently deficient groups were continued on half as much food. Details of the food amounts in each treatment are provided in Table S2. To validate the efficacy of the diet amount treatments, all individuals were weighed on day 15, as individuals in the temporarily deficient food treatments were transitioned to a normal diet. Tadpole microcosms were checked daily and refilled to around 800 ml to maintain water at a constant level. The water of each microcosm was completely refreshed twice weekly. During water refreshment, individual tadpoles were transferred by mesh net to a holding container; wastewater from the assigned microcosm was removed and replaced with clean aged water. Tadpoles were then transferred by net back to the assigned microcosm and fed. Although the *S. bombifrons* used in this experiment are from a population known to produce carnivore morphs (Ledón‐Rettig, 2021), the conditions of the experiment were not conducive to its induction and no carnivores were observed over the course of the experiment.

Upon the first emergence of a forearm, metamorphosing tadpoles were transferred to new microcosms that contained a centimeter of aged water on one side and a sandy “beach” on the other. Upon tail resorption, juveniles were provided with 2cm moistened sand over the entire microcosm and were fed small live crickets dusted with Repti Calcium (Zoo Med) and Herptivite with Beta Carotene (Rep‐Cal). The date of tail resorption (i.e., metamorphosis), weight at metamorphosis, and snout–vent length (SVL) at metamorphosis were recorded. The sand in each juvenile's microcosm was exchanged with fresh sand approximately 6 weeks postmetamorphosis.

### 2.3 Juvenile behavior assays

Approximately 4 weeks following tail resorption, juveniles were assessed for behavioral variation; testing was conducted over multiple days such that all individuals were tested between 26 and 30 days of their date of metamorphosis. Plastic containers (29 cm L × 19 cm W × 17.5 cm H) filled with 7.5 cm of lightly moistened sand were used as our experimental arenas. These conditions are similar to the classic “field assays” used to measure the exploratory and bold or shy behaviors of rodents (Denenberg, 1969). Sand was provided to accommodate the natural tendencies of spadefoot toads: a common response to perceive threats is to bury under the sand (personal observation). Juveniles were carefully placed one per arena, 10 small crickets were added, the researcher (S.R.L.) exited the room, and juvenile behavior was recorded with a digital video camera for a duration of ten minutes. Upon completion of the assay, individuals were returned to their microcosms. Crickets were added to the arenas before each trial as necessary to maintain 10 across each trial. Ultimately, there was a sample size of *n* = 29–33 individuals representing each treatment. Areas explored by juveniles were assessed by the software program ToxTrac (Rodriguez et al., 2018); this program divvies the arena into 28 equal “areas” and tracks the number of areas visited by an individual. Additionally, juvenile behaviors were scored by one observer (S.R.L.) who was blind to the treatment of the individual being assessed. Specifically, the time of initial movement was recorded as latency to move; instances where a juvenile hopped away from a prey item (cricket) were recorded as avoidance behaviors; strikes at prey that resulted in consumption were recorded as successful strikes; strikes at prey that did not result in consumption were recorded as unsuccessful strikes; and the time at which individuals buried under the sand was recorded. Total active time in the arena was calculated as the time between initial movement and either the time of burial or the end of the assay.

Finally, at approximately 7 weeks post‐tail resorption, 15 individuals from each of the six treatment groups were randomly selected and assessed for jumping abilities. Prior to jump assays, the weight of juveniles was taken, and total leg length and SVL were measured via digital caliper. To measure each juvenile's leg length, their leg was gently extended while keeping their body supported. Measurements for the femur, tibiofibular, and foot were made and summed for total leg length. Prior to their jump assay, individuals were rinsed of sand, placed in a dish containing organic food coloring paste, and then transferred to a continuous segment of paper measuring 90 cm length by 26 cm width. If not immediately voluntary, movement was gently encouraged by lightly tapping a pen to the hind quarters. The distance between ten jumps was measured by ruler and recorded. Trials were repeated a total of three times with 5 min of rest between trials.

### 2.4 Stress‐induced and baseline CORT levels

We determine the effects of larval diet type and amount on juvenile baseline and stress‐induced plasma corticosterone (hereafter, CORT) levels at 11–12 weeks after metamorphosis. Half of the frogs from each treatment were subjected to a shaking and confinement stressor (Gabor et al., 2016; Glennemeier & Denver, 2002). Briefly, each individual subjected to the stressor was placed in a microcosm and lightly rocked for 20 min. The bottoms of the microcosms were lined with a moist paper towel to buffer individuals from injury while at the same time keeping them hydrated. Once the stressor was done—or immediately in the baseline stress group—individuals were anesthetized with a ventral topical application of 20% benzocaine gel (Orajel) and then killed by rapid cervical dislocation. Once trunk blood was collected into heparinized capillary tubes, plasma was separated and stored at −80℃. The gonado‐mesonephros complexes were removed and scored to determine each individual's sex; individuals were identified as male, female, or unknown (41.3%, 52.3%, and 6.4% of samples, respectively). The time from handling to blood collection occurred in under 3 min. The final samples sizes for baseline and stress‐induced groups were *n* = 12–16, each (173 samples, total).

Plasma samples were separated into five groups for hormone extraction and enzyme‐linked immunoassay (EIA), randomized by treatment. Solid‐phase steroid extraction was used to purify the samples. Plasma samples were prepared by diluting 10 µl of plasma into 16 ml of ultrapure water. These samples were then run through C18 columns—primed with two runs each of 2 ml methanol and 2 ml ultrapure water—with the vacuum pressure gage maintained around 4 Hg. Subsequently, the columns were rinsed with 4 ml ultrapure water. The CORT steroid sample elution was collected by running 4 ml methanol through the columns after the rinse step. The sample tubes containing the CORT elution were then dried using an Evap‐o‐rac with the samples steeped in a water bath set to 38℃. The samples were resuspended in 20 µl of ethanol plus 380 µl of EIA buffer to achieve a 1:40 dilution. Samples were then individually wrapped in parafilm and stored at −20℃ overnight. Plasma CORT concentrations were then estimated in duplicate with a 480 solid well plate Cayman EIA CORT kit (501320).

### 2.5 Statistics

To determine whether the deficiency treatments affected tadpole growth, the weights of tadpoles were compared at 15 days posthatching. The normality of errors of the weights could not be improved with transformation, so a nonparametric Kruskal–Wallis test was used to determine whether there was a significant difference among normal and deficient groups, and a pairwise Wilcoxon rank sum test was used with Benjamini–Hochberg corrections for multiple comparisons to determine specific significant differences between all groups.

To further determine whether larval diet type or amount influenced juvenile growth across development, weights at metamorphosis and beyond metamorphosis by 4 and 7 weeks were compared among treatments. Weights at each of these time points were natural log‐transformed to meet the conditions of normality before they were assessed in separate ANOVAs with diet type, diet amount, and their interaction as explanatory variables. Additionally, to assess whether larval diet type or amount influenced growth rate, individual growth rates across development were derived as changes in mass divided by the number of days in each time interval (from hatching to 15 days, from 15 days to metamorphosis, from metamorphosis to 4 weeks postmetamorphosis, and from 4 weeks postmetamorphosis to 7 weeks postmetamorphosis). Two of the growth rates (early larval and 7 weeks postmetamorphosis) did not meet the conditions of normality and were assessed using separate nonparametric Kruskal–Wallis tests, followed by post hoc analyses with Benjamini–Hochberg corrections. The late larval growth rate nearly met the conditions of normality and was assessed in an ANOVA with diet type, diet amount, and their interaction as explanatory variables; it was also assessed using a nonparametric Kruskal–Wallis test, and the results were qualitatively identical to those from the ANOVA. The 4 weeks postmetamorphosis growth rate met the conditions of normality and was assessed in an ANOVA with diet type, diet amount, and their interaction as explanatory variables.

To determine whether developmental period itself was influenced by diet type and amount, time to metamorphosis was natural log‐transformed to improve the conditions of normality before it was assessed in an ANOVA with diet type, diet amount, and their interaction as explanatory variables. Since natural log‐transformation did not completely rectify the normality of developmental time errors, it was also assessed using a nonparametric Kruskal–Wallis test, followed by a post hoc analysis with Benjamini–Hochberg corrections, and the results were qualitatively identical to those from the ANOVA. Here and in all ANOVAs above, insignificant factors were removed by backward elimination until only significant explanatory factors remained, and Tukey's HSD tests were used to determine significant differences between levels of explanatory factors while controlling for multiple comparisons. In cases where there were interacting factors, the R package *lsmeans* (Lenth, 2016) was used to determine significant differences between groups while controlling for multiple comparisons using the Benjamini–Hochberg procedure.

To determine whether larval diet type and amount influenced juvenile behaviors, we assessed the following variables: latencies to move (in seconds), the strike efficiency (measured as the proportion of successful prey strikes), the proportion of areas explored in the arena, the number of avoidance behaviors, and the total active time in the arena measured as the time between initial movement and the time of burial or completion of the assay. After natural log‐transformation, latency to move met the conditions of normality and was therefore modeling using ANOVA. Avoidance behaviors were modeled with a generalized linear model (GLM) using a Poisson distribution for count data (O'Hara & Kotze, 2010). Strike efficiency (successful strikes and unsuccessful strikes) and the proportion of areas explored (areas explored and areas not explored) were modeled as binary variables in separate GLMs using binomial distributions. The total time spent exploring the arena was left‐skewed and was therefore modeled using a GLM with a gamma distribution. For all behavioral models, diet type, diet amount, and their interactions served as explanatory variables, and covariates were an individual's sex, SVL, and natural log‐transformed scaled mass index at the time of the assay (4 weeks postmetamorphosis). The scaled mass index is an indicator of body condition that specifically provides an estimate of body mass as if all individuals were the same length (Peig & Green, 2009). For all GLMs, we compared models with and without terms with a likelihood ratio test to determine whether they improved the fit of the model, and those that did not were removed in a stepwise fashion. In cases where there were interacting factors, *lsmeans* was used determine significance between groups while controlling for multiple comparisons using the Benjamini–Hochberg procedure.

To determine whether the larval environment influenced relative leg length of the subset of individuals used for the jump performance test, we used residual leg length (calculated by regressing leg lengths on SVL at 7 weeks) as a response variable in an ANOVA with the explanatory variables diet type, diet amount, and their interaction. Additionally, average jump length and longest jump length (both normally distributed) were assessed in separate ANOVAs with the explanatory variables diet type, diet amount, and their interaction, and covariates were an individual's sex, residual leg length, SVL, and natural log‐transformed scaled mass index at the time of the assay (7 weeks postmetamorphosis).

Plasma CORT concentrations were natural log‐transformed before being used as a response variable. Separate ANOVAs were used for each diet amount. Explanatory variables included diet type, stress level, and their interaction, as well as sex, SVL at 4 weeks postmetamorphosis, and natural log‐transformed scaled mass index at 4 weeks postmetamorphosis (this time point was used because it was the last time at which the masses and SVLs of all individuals were recorded) as covariates. Survival for the experiment was assessed using a Cox regression for survival analysis using the R package *survival* (Therneau, 2020); further details are provided in the Supporting Information; however, no significant differences in survival between treatments were detected.

## 3 RESULTS

### 3.1 Spadefoot larvae exhibit catch‐up and compensatory growth

**FIGURE 2:**
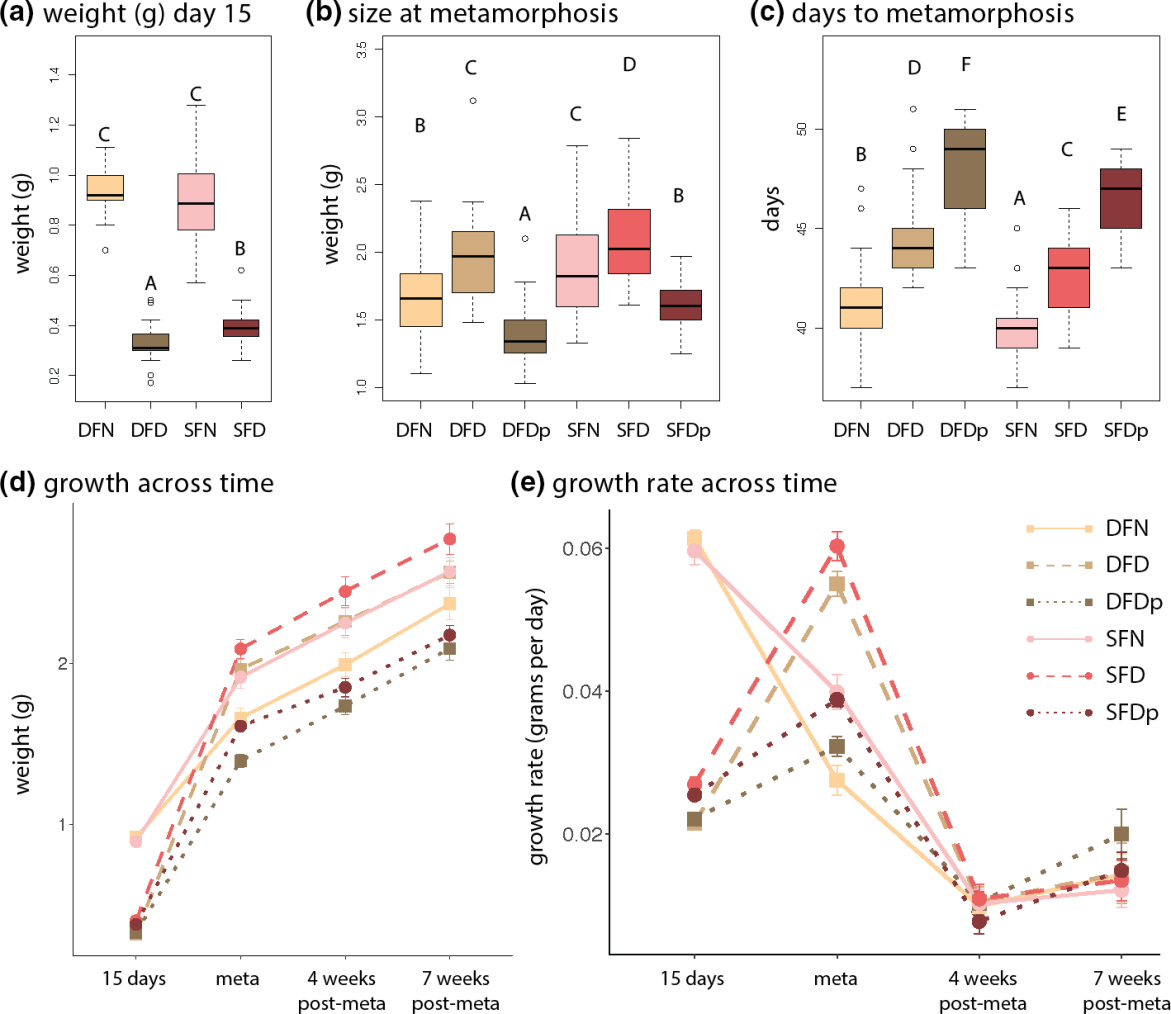
Larvae that experience a temporary nutritional deficit experience catch‐up and compensatory growth. Larvae fed a deficient diet for 15 days after hatching experienced significantly attenuated growth compared to those fed a normal diet (a). By metamorphosis, individuals fed a deficient diet that were transitioned to a normal diet at 15 days had significantly surpassed in mass larvae that had been fed a consistently normal diet (b). These differences in size persisted for 7 weeks postmetamorphosis (d). The recovery of individuals in the temporarily deficient group was in part due to both a significantly extended growth period (catch‐up growth; c) and significantly accelerated growth rate (compensatory growth; e). Lines within boxplots are median values and whiskers extend to the most extreme data points that are not outliers (open circles). Letters indicate significant differences between groups (DFN and SFN: normal detritus‐ and shrimp‐fed; DFD and SFD: temporarily deficient detritus‐ and shrimp‐fed; DFDp and SFDp: permanently deficient detritus‐ and shrimp‐fed). Error bars in the line graphs indicate standard error for each treatment

Full statistics tables are provided in Table S3. Larval growth at 15 days was significantly influenced by nutritional amount and diet (*H*_(3)_ = 134.57, *p* < 0.001; Figure 2a). Specifically, larvae that were either shrimp‐ or detritus‐fed at normal amounts attained similar sizes (*Z* = 0.95, *p* = 0.34), but both were significantly larger than their shrimp‐ or detritus‐fed counterparts fed at deficient amounts (*Z* = 7.59, *p* < 0.001 for detritus‐fed and *Z* = 7.75, *p* < 0.001 for shrimp‐fed). Individuals fed a deficient amount of shrimp were significantly larger than individuals fed a deficient amount of detritus (*Z* = 5.27, *p* < 0.001). The raw data for weight at metamorphosis by developmental time are supplied in Figure S1.

Both diet type (*F*_(1,180)_ = 24.23, *p* < 0.001) and amount (*F*_(2,180)_ = 51.67, *p* < 0.001), but not their interaction, significantly explained variation in individual weight at metamorphosis. Specifically, individuals fed a temporarily deficient diet were heavier than those fed a normal (*p*_adj_ < 0.001) or permanently deficient diet (*p*_adj_ < 0.001), and shrimp‐fed individuals grew more on average than detritus‐fed individuals (*p*_adj_ < 0.001; Figure 2b). This pattern could be in part explained by the extended larval period of those in the temporarily deficient diet: diet type (*F*_(1,180)_ = 22.38, *p* < 0.001) and amount (*F*_(2,180)_ = 158.18, *p* < 0.001), but not their interaction, significantly explained variation in developmental time to metamorphosis. Specifically, shrimp‐fed individuals took less time to develop than those who were detritus‐fed (*p*_adj_ < 0.001) and individuals fed a normal diet took less time to develop than those fed either temporarily or permanently deficient diets (*p*_adj_ < 0.001, Figure 2c). The additional growth allowed by the extended larval period in individuals experiencing a temporarily deficient diet is consistent with a pattern of catch‐up growth.

These differences in growth due to diet type (*F*_(1,179)_ = 9.22, *p* < 0.001) and amount (*F*_(2,179)_ = 28.75, *p* < 0.001) persisted 4 weeks postmetamorphosis, with juveniles from shrimp‐fed larvae still heavier than those from detritus‐fed larvae (*p*_adj_ = 0.003) and juveniles from the temporarily deficient diet still heavier than those from the normal diet (*p*_adj_ = 0.01). Further, diet type (*F*_(1,174)_ = 7.01, *p* = 0.01) and amount (*F*_(2,174)_ = 14.65, *p* < 0.001) differences persisted 7 weeks postmetamorphosis, with juveniles from shrimp‐fed larvae still heavier than those from detritus‐fed larvae (*p*_adj_ = 0.01) and juveniles from the temporarily deficient diet heavier than those from the normal diet (*p*_adj_ = 0.04; Figure 2d).

Finally, growth rates—measured as the change in weight over the days of a defined developmental period—varied by treatment across time (Figure 2e). During early larval development, those fed deficient diets had a significantly lower growth rate than those fed normal diets, mirroring their weights at 15 days (Table S3). During late larval development (15 days posthatching to metamorphosis), differences in growth rates varied by diet type (*F*_(1,180)_ = 27.41, *p* < 0.001) and amount (*F*_(2,180)_ = 103.35, *p* < 0.001); specifically, shrimp‐fed larvae had a greater growth rate than detritus‐fed larvae (*p*_adj_ < 0.001) and larvae fed a temporarily deficient diet had a greater growth rate than either those fed a normal or permanently deficient diet (both contrasts, *p*_adj_ < 0.001). These differences, however, disappeared at 4 weeks and 7 weeks metamorphosis, with neither larval diet type nor amount significantly influencing growth rate. Thus, the differences in individuals’ sizes at metamorphosis can partially be explained by accelerated growth during late larval development, a pattern of compensatory growth.

### 3.2 Juvenile behaviors depend on larval diet type and amount

**FIGURE 3:**
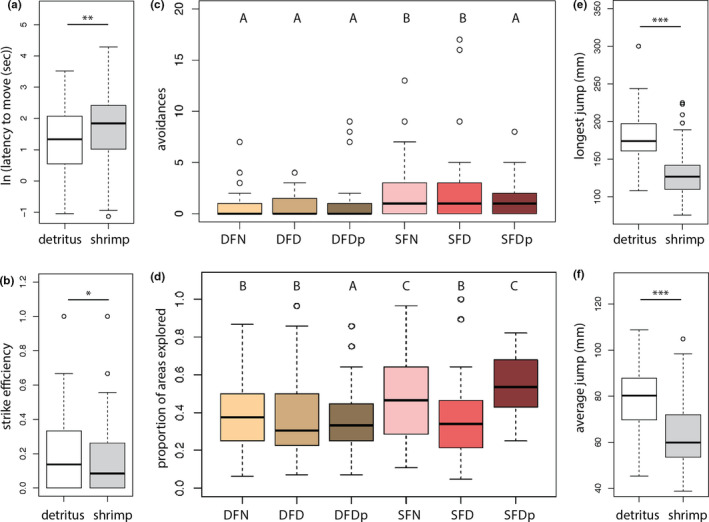
Juvenile behavioral traits are influenced by larval diet type and amount. Juveniles derived from shrimp‐fed larvae had significantly (**, *p* = 0.01) longer latencies to move (a) and (*, *p* = 0.05) poorer strike efficiencies (b) than juveniles derived from detritus‐fed larvae. Juveniles derived from shrimp‐fed larvae avoided prey items significantly (*p* < 0.001) more, although this effect disappeared if their larval diet was permanently deficient (c), and juveniles derived from shrimp‐fed larvae explored a significantly (*p* = 0.009) greater proportion novel areas, although this effect disappeared if they experienced a temporary nutritional stressor as larvae (d) (DFN and SFN: normal detritus‐ and shrimp‐fed; DFD and SFD: temporarily deficient detritus‐ and shrimp‐fed; DFDp and SFDp: permanently deficient detritus‐ and shrimp‐fed; letters indicate significant differences between groups). Juvenile jumping abilities were also influenced by larval diet, with significantly longer maximum (e) and average jumps (f) in juveniles derived from detritus‐versus shrimp‐fed larvae. Lines within boxplots are median values and whiskers extend to the most extreme data points that are not outliers (open circles)

Postmetamorphosis, an individual's latency to move in a novel environment was significantly influenced by larval diet type with those fed shrimp as larvae taking longer to move than those fed detritus as larvae (*F*_(1,181)_ = 7.35, *p* = 0.01; Figure 3a). Juvenile strike efficiency was significantly lower in juveniles fed shrimp as larvae relative to those fed detritus as larvae (*χ* ^2^ = 3.96, *p* = 0.05; Figure 3b). Juvenile avoidance behavior was influenced by an individual's sex (*χ* ^2^ = 20.98, *p* < 0.001), length (*χ* ^2^ = 8.50, *p* = 0.004), and the interaction between their diet type and amount (*χ* ^2^ = 8.25, *p* = 0.02); post hoc tests revealed that individuals fed shrimp as larvae had significantly more avoidance behaviors only if they were derived from a normal (*Z* = 2.32, *p*_adj_ = 0.04) or temporarily deficient (*Z* = 2.70, *p*_adj_ = 0.02; Figure 3c) diet, and males had significantly more avoidance behaviors than females (*Z* = 2.86, *p*_adj_ = 0.006; Fig S4a). Total activity time was not significantly influenced by any of the variables assessed. The proportion of areas explored was significantly influenced by and individual's sex (*χ* ^2^ = 33.42, *p* < 0.001), length (*χ* ^2^ = 98.56, *p* < 0.001), and scaled mass index (*χ* ^2^ = 58.10, *p* < 0.001) and the interaction between their larval diet type and amount (*χ* ^2^ = 22.11, *p* < 0.001; Figure 3d). Specifically, post hoc tests revealed that—only in individuals fed shrimp as larvae—juveniles derived from the temporarily deficient larval diet group explored a lower proportion of areas than those derived from the normal (*Z* = 2.95, *p*_adj_ = 0.007) or permanently deficient larval diet groups (*Z* = 2.41, *p*_adj_ = 0.02), and males explored significantly more areas than females (*Z* = 5.14, *p*_adj_ < 0.001; Fig S4b).

Neither diet type nor amount significantly described variation in relative leg length. However, juveniles who were detritus‐fed as larvae jumped 35% farther on their longest hop (176.78 ± 37.22 vs. 130.78 ± 35.72, mean ± *SD*) and 22% on their averaged hops (78 ± 16.19 vs. 64.26 ± 16.60, mean ± *SD*) than those who were shrimp‐fed as larvae (Figure 3e,f). The effect of diet on both longest and average jump was significant (*F*_(1,88)_ = 35.77 and *F*_(1,84)_ = 17.03; both *p* < 0.001). In neither case did an individual's larval diet amount, relative leg length, overall length (SVL), or scaled mass index influence jumping performance. For average jump, sex was also a significant explanatory factor (*F*_(2,84)_ = 35.77; *p* = 0.04); however, a post hoc test did not reveal significant differences between the three levels (male, female, and unknown); removing individuals with unknown sex (*n* = 4) from the model rendered the effect of sex insignificant.

### 3.3 Baseline and stress‐induced CORT levels depend on larval diet type and amount

**FIGURE 4:**
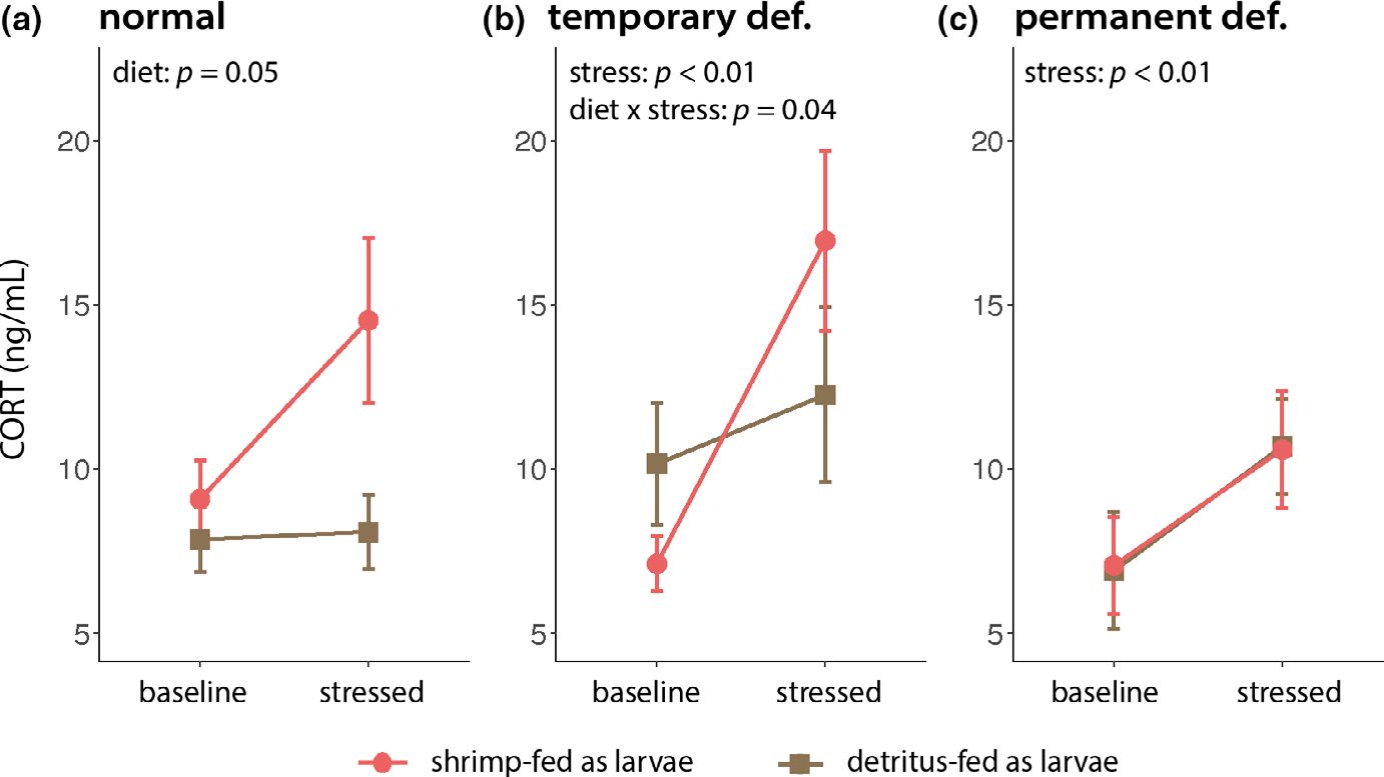
Juvenile stress axis reactivity varies by nutritional amount and diet type. Juveniles that were shrimp‐fed as larvae had higher baseline and stress‐induced CORT levels than those that were detritus‐fed as larvae when larval nutritional amounts were normal (a). Juveniles that were shrimp‐fed as larvae had higher stress‐induced CORT levels than those that were detritus‐fed as larvae when larvae experienced a temporary nutritional deficit (b). Finally, diet had no effect on baseline or stress‐induced CORT levels when larval nutritional amounts were permanently deficient (c). Untransformed mean values and their standard errors are presented as shapes (circles and squares) and bars, respectively

Among juveniles fed a normal amount of food as larvae, those that consumed shrimp as larvae had significantly higher (*F*_(1,56)_ = 4.14, *p* = 0.05) CORT levels across baseline and stress‐induced treatments (Figure 4a). Among juveniles that experienced a temporary early‐life nutritional deficiency, not only did the stress treatment result in significantly higher CORT levels (*F*_(1,50)_ = 9.32, *p* < 0.01), larval diet type significantly interacted with the stress treatment (*F*_(1,50)_ = 4.38, *p* = 0.04; Figure 4b); specifically, a post hoc comparison revealed that juveniles derived from shrimp‐fed larvae had higher stress‐induced relative to baseline CORT levels (*p*_adj_ = 0.003). Finally, among juveniles that experienced permanent early‐life nutritional deficiency, the stress treatment produced significantly higher CORT levels (*F*_(1,57)_ = 11.29, *p* < 0.01), but this effect did not depend on their larval diet type (Figure 4c). Neither an individual's scaled mass index nor sex had a significant effect on CORT levels.

## 4 DISCUSSION

Although the ability to utilize diverse food resources is a common feature of animals, it is not well understood how early‐life nutritional deficiency interacts with diet type to shape phenotypes later in life. Here, we have addressed this question using a spadefoot toad species whose larvae can consume either detritus or, as an evolutionary novelty among anuran larvae, a diet of shrimp. We found that the derived larval diet and nutritional stress have sometimes independent and sometimes interacting effects on juvenile prey‐capture abilities, jumping performances, and glucocorticoid responses. Our results suggest that considering ecologically relevant variation in diet type is critical to understanding the effects of early‐life nutritional stress on later life behaviors and physiologies.

Here, individuals that were switched from limited to normal nutritional conditions caught up to and surpassed individuals consistently fed a normal diet in growth by both accelerating their growth rate (i.e., compensatory growth) and extending their larval period (i.e., catch‐up growth; Dahl et al., 2012; Orizaola et al., 2014). Thus, juveniles that had experienced a temporary nutritional deficit as larvae had modified behaviors and physiologies, even after recovering in body size. This finding corroborates widespread evidence that interactions between early and late developmental nutritional environments can influence later life phenotypes (Gluckman et al., 2005). Uniquely, however, the effects of the interactions between nutritional environments in our study were only seen in the context of a specific larval diet type. Specifically, juvenile exploratory behavior was modified by a temporary early‐life nutritional deficit in individuals derived from shrimp‐fed, but not detritus‐fed, larvae. Likewise, juveniles who experienced a temporary nutritional deficit *and* were fed shrimp as larvae exhibited elevated glucocorticoid responses; differences in stress axis reactivity between the diets were not apparent in juveniles that had experienced a consistently ample or consistently deficient larval diet. Although the ramifications of exploratory juvenile behaviors or elevated glucocorticoid responses of *S. bombifrons* in its native habitats are unknown, such phenotypic variation is likely to have ecological and evolutionary consequences (Réale et al., 2010; Sloan Wilson et al., 1994; Vitousek et al., 2018; Wolf & Weissing, 2012).

Additionally, the novel larval diet of shrimp had effects on juvenile behaviors that were independent of nutritional amount. Regardless of the amount of larval diet provided, juveniles raised as shrimp‐fed larvae had worse prey‐capture efficiencies and shorter jump lengths (effects that were not mediated by relative leg length nor overall size). It is plausible that these modifications, induced by larval diet type, are costly to *S. bombifrons* juveniles as they attempt to secure food or evade threats, particularly during this life stage. It is precisely during the juvenile stage where growth is critical for avoiding desiccation (Goater, 1994; Newman & Dunham, 1994; Pfennig, 1992) and where jumping can aid small individuals that are at risk for predation (Garland & Losos, 1994; Irschick & Garland, 2001).

Although studies have documented the influence of early‐life nutritional restriction on adult baseline and stress‐induced CORT levels (Hu et al., 2008; Monaghan, 2008; Warne & Crespi, 2015), our study provides new insight into how diet type can modify these carryover effects (see also Kitaysky et al., 2001). Specifically, among juveniles that experienced ample nutritional conditions as larvae, those that were shrimp‐fed as larvae had higher overall CORT levels than those that were detritus‐fed as larvae. While stress‐induced glucocorticoids generally serve an important function by initiating physiological and behavioral changes to help an individual cope with an environmental stressor (Romero, 2004; Sapolsky et al., 2000; Wingfield et al., 1998), higher titers of circulating glucocorticoids can be harmful to an individual's health (Khulan & Drake, 2012; Sapolsky et al., 1986). Whether the higher baseline CORT levels possessed by juveniles derived from shrimp‐fed larvae reflect chronically unhealthful glucocorticoid levels is unclear. Future studies will determine whether these elevated CORT levels impact survival, reproduction, or even offspring phenotypes, in natural populations of *S. bombifrons*.

In comparison with juveniles who as tadpoles were fed an ample shrimp diet—who had higher overall CORT levels—juveniles who, as tadpoles, both were fed shrimp and experienced a temporary nutritional deficit exhibited higher stress‐induced CORT levels; that is, they exhibited higher stress reactivity. How was juvenile stress axis reactivity influenced by larval diet? Across animal systems, stress hormones that are experimentally elevated during early development cause the HPA (hypothalamic–pituitary–adrenal) axis to be “reprogrammed” such that adults have modified stress axis activity (Harris & Seckl, 2011; Hu et al., 2008; Pravosudov & Kitaysky, 2006; Spencer et al., 2009). One hypothesis for how this reprogramming occurs is that higher stress hormone levels during development cause the expression of corticosteroid receptors—glucocorticoid and mineralocorticoid receptors—to be permanently lowered, and in turn compromise the sensitivity of the stress axis’ negative feedback mechanism, leading to higher stress‐induced CORT levels (Seckl & Meaney, 2004). A previous study using *S. bombifrons* found that shrimp‐fed and detritus‐fed larvae had indistinguishable CORT levels (Ledón‐Rettig et al., 2009), although CORT titers were measured only as baseline levels during a single, relatively early point during development. Thus, additional studies are needed to determine whether larval stress hormones themselves—or some other physiological mechanism—couples early‐life plasticity in response to diet type and nutritional stress to the stress axis reactivity of juveniles.

Although several studies have characterized the carryover effects of early‐life diets that differ in quality or amount (reviewed in Harrison et al., 2011), here we have addressed the effects of early‐life diets that represent distinct dietary niches. Previous studies have found that tadpoles consuming animal‐based or protein‐rich diets generally grow better, develop faster, and enjoy higher survival than those consuming plant‐based diets (Álvarez & Nicieza, 2002; Crump, 1990; Kupferberg, 1997; Steinwascher & Travis, 1983; Venesky et al., 2012). Consistent with these findings, *S. bombifrons* individuals in this study that consumed a shrimp diet as larvae metamorphosed more quickly and at a larger size than those derived from detritus‐fed larvae. Indeed, it is for these reasons that spadefoot carnivores in nature—which specialize on shrimp—are sometimes the only individuals to escape particularly ephemeral ponds, especially when competition is intense (Pfennig, 1992). Yet despite its benefit to larval growth and developmental speed, the consumption of shrimp resulted in latent effects on juvenile behaviors and glucocorticoid responses that potentially temper these benefits. In order to understand whether diet‐induced carryover effects in the spadefoot system really do recalibrate an individual's fitness, further studies must address how adults survive and reproduce after leaving the ponds, and over different environmental conditions.

## 5 CONCLUSIONS

The effects of early‐life conditions on adult phenotypes are broadly relevant to several biological disciplines, from ecology and evolution to epidemiology and conservation biology. This study contributes to our understanding of such carryover effects by demonstrating that early‐life nutritional stress and diet type can interact to influence later life behaviors and physiologies. We have approached this question in American spadefoot toads, a clade that possesses species and populations that have evolved varying levels of developmental plasticity from subtle to extraordinary (Ledón‐Rettig & Pfennig, 2011). By using spadefoots in a comparative framework, future studies will seek to understand how the types of carryover effects identified in this study influence individual fitness in nature, how they evolve, and how they influence the evolution of plasticity itself.

## CONFLICT OF INTEREST

The authors have no competing interests.

## AUTHOR CONTRIBUTIONS

**Cristina C. Ledón‐Rettig:** Conceptualization (lead); Formal analysis (lead); Funding acquisition (lead); Investigation (equal); Methodology (equal); Project administration (lead); Visualization (lead); Writing‐original draft (lead); Writing‐review & editing (equal). **Sarah R. Lagon:** Conceptualization (supporting); Data curation (lead); Investigation (equal); Methodology (equal); Writing‐original draft (supporting); Writing‐review & editing (equal).

## Supporting information

Supplementary Material

Table S3

## Data Availability

The data reported in this paper have been deposited in the Dryad repository (https://doi.org/10.5061/dryad.fbg79cnvc).
