# Supplementary material for: A novel larval diet interacts with nutritional stress to modify juvenile behaviors and glucocorticoid responses
Source: Ecol Evol. 2021 Jul 28;11(16):10880–91. doi: 10.1002/ece3.7860 (PMC8366881; doi:10.1002/ece3.7860)
Supplement: Supplementary file 1 — Supplementary Material [file ECE3-11-10880-s002.docx]

**Supporting Information for: A novel larval diet interacts with dietary stress to modify juvenile behaviors and glucocorticoid responses.** Cris C. Ledón-Rettig and Sarah R. Lagon

**(Table S3 is provided as an excel workbook)**

**Figure S1 |** Raw data of weight at metamorphosis across time to metamorphosis. All treatments are presented in the left panel, and treatments grouped by diet amount are presented in the right panel.


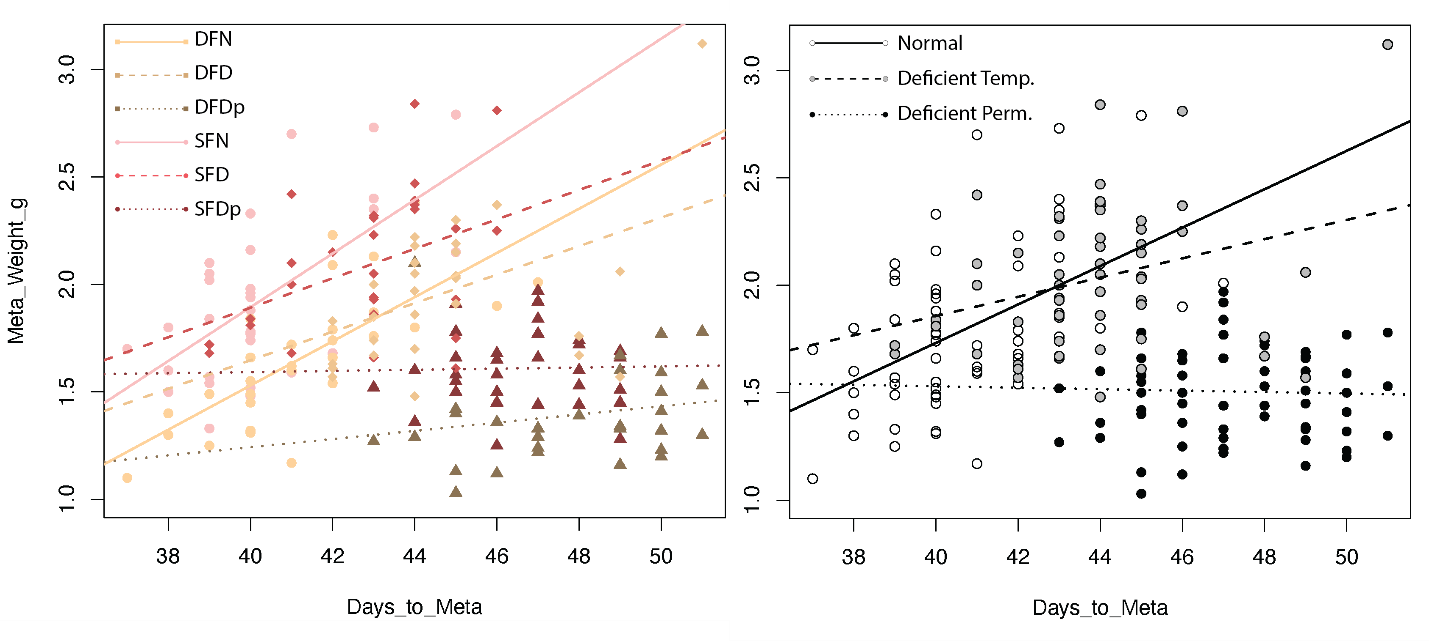


**Survival Analysis |** To determine whether larval diet type or amount influenced pre- or post-metamorphic survival, we used a Cox regression for survival analysis using the R package *survival* (Therneau, 2020). The Cox regression is a nonparametric method for investigating the effect of several variables, such as the ones manipulated in this study, upon the time a specified event, such as death, takes to happen. A visual assessment suggested that there may be significant differences in survival owed to the diet type and amount treatments in our study:

**Figure S2 |** Survival by diet type and amount


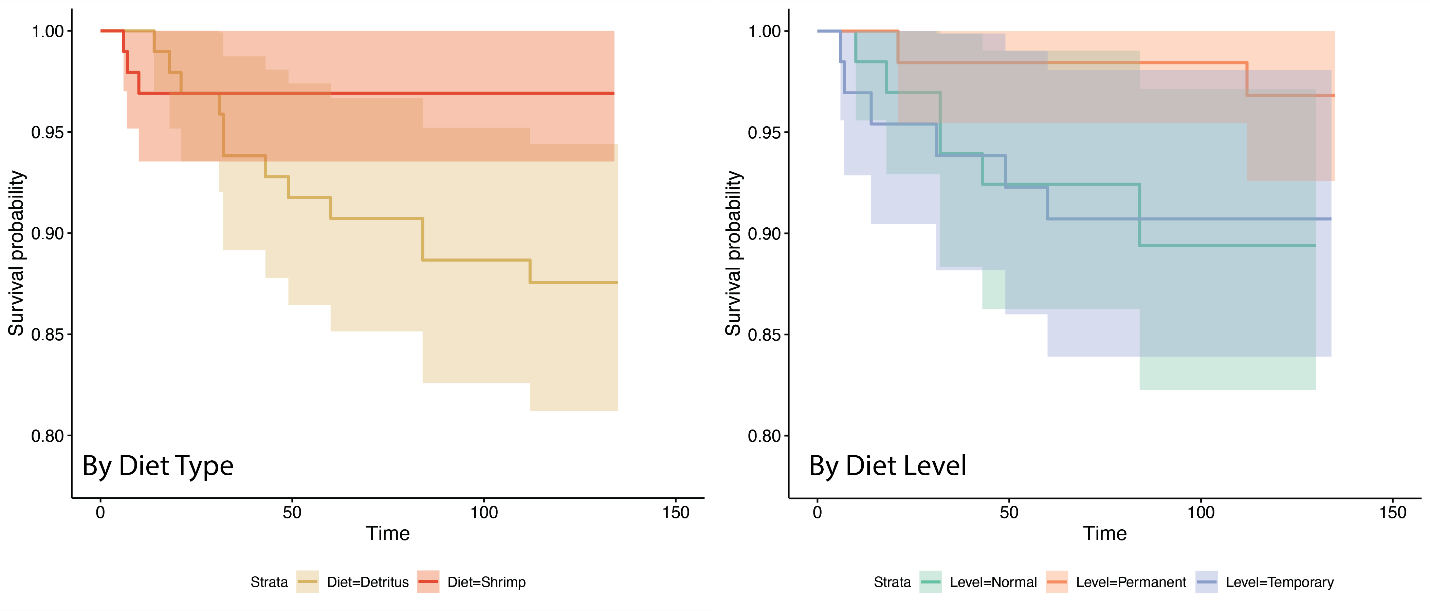


However, the Cox method assumes that the effects of the predictor variables upon survival are constant over time and additive in scale, and a preliminary analysis (the cox.zph function, which correlates scaled Schoenfeld residuals with time) revealed that the proportional hazards assumption was violated. We therefore proceeded by assessing each of three time-bins: (i) early larval development to 15 days; (ii) late larval development to metamorphosis; and (iii) post metamorphic development until the termination of the study. Early larval development was defined as the interval spanning the initiation of diet treatments to 15 days later. Late larval development was defined as the interval spanning the 15 days after the initiation of diet treatments to an individual’s foreleg emergence date. Post-metamorphosis was defined as the interval spanning an individual’s foreleg emergence date to the day they were euthanized. These time bins coincided with major transitions in the study, such as the transition of the temporarily deficient group to a normal diet, and the transition of all larval groups to their juvenile state. When assessed separately, the proportional hazards assumption was met in each time frame.

**Figure S3 |** Survival by diet across developmental periods


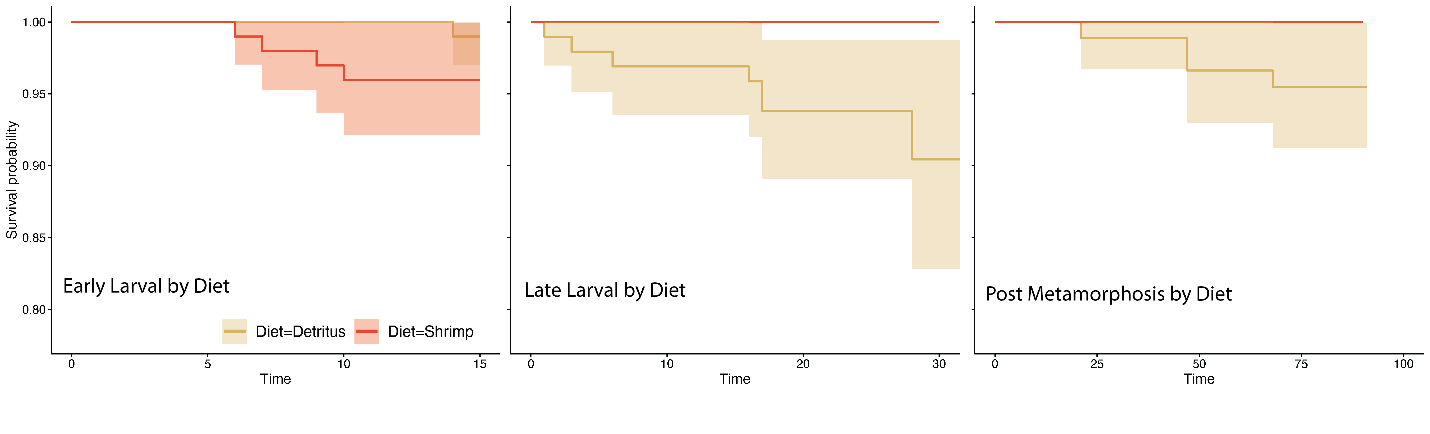


We determined the statistical significance of models by comparing their fits to reduced models with a log-likelihood ratio test. The Cox final reduced model results are reported, below. The regression coefficients represent the log change in the hazard function per unit increase of the predictor variable (*i.e.*, a negative hazard rate indicates increased survival). In the reduced, best models, none of the predictor variables made a significant difference in survival.

**Figure S4 |**Avoidance and exploratory behaviors by sex (F: female; M: male; U: unknown). Letters indicate significant differences between groups.


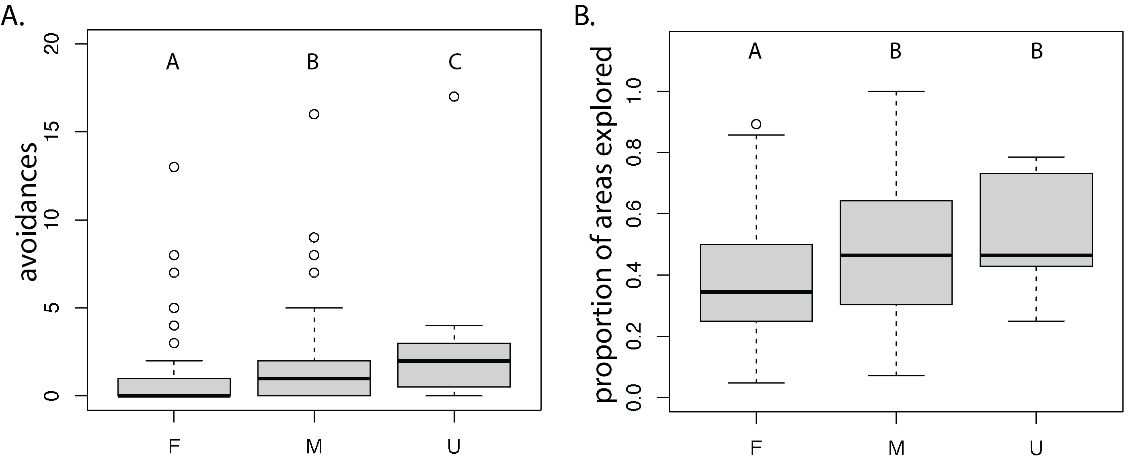


**Table S1** | Temperatures of the room where animals were housed and experiments took place

**Table S2** | Diet amounts across treatments for the duration of the experiment.
